# Prognostic predictors of radical resection of stage I-IIIB non-small cell lung cancer: the role of preoperative CT texture features, conventional imaging features, and clinical features in a retrospectively analyzed

**DOI:** 10.1186/s12890-023-02422-7

**Published:** 2023-04-14

**Authors:** Xingxing Zheng, Rui Li, Lihua Fan, Yaqiong Ge, Wei Li, Feng Feng

**Affiliations:** 1 Department of Radiology, Xi’an Jiaotong University, Xi’an, 710049 China; 2 Department of Radiology, Baoji Central Hospital, Baoji, 721000 China; 3 Department of Radiology, Affiliated Tumor Hospital of Nantong University, No. 30 Tongyangbei Road, Tongzhou District, Nantong, 226361 China; 4 Department of Radiology, Affiliated Hospital of Shaanxi University of Traditional Chinese Medicine, Xianyang, 712000 China; 5 GE Healthcare China, Shanghai, 210000 China

**Keywords:** CT, Non-small cell lung cancer (NSCLC), Survival, Texture analysis, Prognostic

## Abstract

**Background:**

To investigate the value of preoperative computed tomography (CT) texture features, routine imaging features, and clinical features in the prognosis of non-small cell lung cancer (NSCLC) after radical resection.

**Methods:**

Demographic parameters and clinically features were analyzed in 107 patients with stage I-IIIB NSCLC, while 73 of these patients received CT scanning and radiomic characteristics for prognosis assessment. Texture analysis features include histogram, gray size area matrix and gray co-occurrence matrix features. The clinical risk features were identified using univariate and multivariate logistic analyses. By incorporating the radiomics score (Rad-score) and clinical risk features with multivariate cox regression, a combined nomogram was built. The nomogram performance was assessed by its calibration, clinical usefulness and Harrell’s concordance index (C-index). The 5-year OS between the dichotomized subgroups was compared using Kaplan–Meier (KM) analysis and the log-rank test.

**Results:**

Consisting of 4 selected features, the radiomics signature showed a favorable discriminative performance for prognosis, with an AUC of 0.91 (95% CI: 0.84 ~ 0.97). The nomogram, consisting of the radiomics signature, N stage, and tumor size, showed good calibration. The nomogram also exhibited prognostic ability with a C-index of 0.91 (95% CI, 0.86–0.95) for OS. The decision curve analysis indicated that the nomogram was clinically useful. According to the KM survival curves, the low-risk group had higher 5-year survival rate compared to high-risk.

**Conclusion:**

The as developed nomogram, combining with preoperative radiomics evidence, N stage, and tumor size, has potential to preoperatively predict the prognosis of NSCLC with a high accuracy and could assist to treatment for the NSCLC patients in the clinic.

**Supplementary Information:**

The online version contains supplementary material available at 10.1186/s12890-023-02422-7.

## Background

Currently, lung cancer has become the most common malignant tumor worldwide with increasing morbidity and mortality year by year [1, 2]. Non-small cell lung cancer (NSCLC) is the major histological type of lung cancer that accounts for 75% to 85% of the total cases [3]. The outcomes of patients with locally advanced NSCLC remained poor in recent years with a median survival of 12–23.2 months [4]. Therefore, better prognostic tools are needed to avoid unnecessary and potentially harmful treatments at the end of life and to provide specific treatments to improve the quality of life of advanced patients.

Computed tomography (CT) is widely used for the clinical staging of NSCLC, diagnosis, treatment guidance, etc. [5, 6]. However, information provided by standard imaging modalities usually refers to some simple traits, such as tumor size, and gross shape. Recently, texture analysis based on CT imaging has gained increasing attention. It is one of the methods that can reflect some of the tumor's internal components and the heterogeneity of various tumors [7, 8]. This method extracts a large amount of useful data or information from the image to mathematically detect local spatial changes in pixel intensity [9, 10]. Moreover, texture analysis basically contains the gray level cooccurrence matrix (GLCM) and histogram analysis. Compared to simple visual analysis, texture analysis also has the advantage of quantifying tumor heterogeneity [11]. At present, the texture analysis performed on CT images of lung tumors has identified parameters reflecting tumor heterogeneity that are associated with advanced disease [12]. Moreover, previous reports suggest that the texture features of tumors may be important predictors of the histological subtypes [13], lymph node metastasis [14] and preoperative staging [15] in lung cancers.

The preliminary step is to determine whether tumor heterogeneity assessed by CT texture analysis is a predictor of survival for NSCLC patients. Therefore, the aim of this study was to develop a novel approach for the noninvasive and individualized assessment of the overall survival (OS) in patients with NSCLC. A practical nomogram that incorporated the radiomic signature and other clinicopathological characteristics was also developed for the prediction of OS in patients with NSCLC.

## Methods

### Patients

The electronic medical records of 107 patients diagnosed with NSCLC from March 2004 to June 2014 were collected from the medical institution system and retrospectively analyzed in one center. This study was approved by the Ethics Committee of the Cancer Hospital of Nantong University and the requirement for informed consent from the patients was waived.

**Fig. 1:**
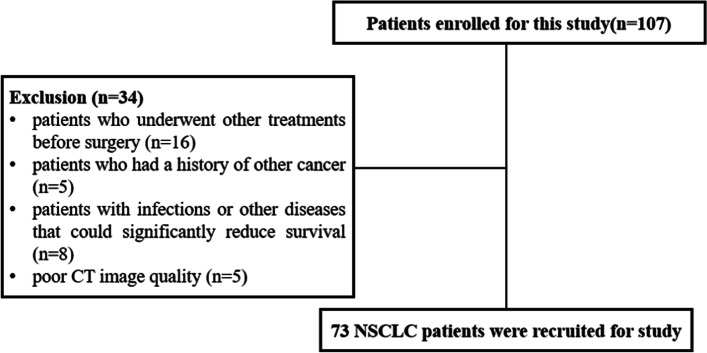
Flow chart of patient selection

The inclusion criteria were as follows: (1) pathological confirmation of NSCLC; (2) patients who underwent CT examination within 1 month before surgery; and (3) detailed medical records and test results. The exclusion criteria were as follows: (1) patients who underwent other treatments before surgery; (2) patients who had a history of other cancer; (3) patients with infections or other diseases that could significantly reduce survival; and (4) poor CT image quality. The study workflow diagram of patient selection is shown in Fig. 1. Finally, 73 patients were included in the study cohort.

### Clinicopathological characteristics

For each patient, we collected clinicopathological characteristics, including age, sex, tumor diameter (> 5 cm or < 5 cm), T stage, N stage, lobes, burr pleural depression, leaflet, histology, and postoperative adjuvant therapy. Subgroup analysis was performed to explore the impacts of these factors on patient survival. Staging was performed according to the latest National Comprehensive Cancer Network guidelines (version 1.2019).

### Follow-up and prognostic assessment

We obtained the survival information of these patients through telephone inquiries, medical insurance records and death certificates. OS was calculated as the period from the initial operation to death or the last follow-up.

### CT acquisition

All CT images were acquired by a Siemens 64-slice spiral CT scanner (Siemens Medical Systems, Germany) with the following procedure. The patient was placed in a supine position with hands raised and instructed to take a deep breath for scanning. The scan range included the entire lung. The followed scanning parameters were used: field of view, 300 mm; matrix, 512 × 512; tube current, 250 mAs; tube voltage, 120 kV; and reconstruction layer thickness, 1 mm. The nonionic iodine contrast agent iohexol (containing 300 mg of iodine per milliliter; 80 to 100 mL) was injected through the elbow vein at a rate of 3.5 mL/s. The scans were performed during the arterial and venous phases. The arterial phase started at 25 s post-injection and the venous phase started at 30 s after the completion of the arterial phase. To improve the repeatability, this study chose the same CT acquisition and reconstruction parameters (slice thickness of 1 mm and standard kernel), and the reconstructed scan images were automatically transferred to the PACS system.

### Image analysis

Two radiologists with 5 and 7 years of chest imaging experience were responsible for tumor segmentation. Thoracic unenhanced CT images with a 1 mm thickness were imported into ITK-SNAP software (a free/open source software, version 4.3.2 (www.itksnap.org) to delineate the region of interest (ROI) of NSCLC. The primary three-dimensional (3D) ROI was manually delineated slice-by-slice. Segmentation was started from the mediastinum window setting (width: 450 HU; level: 50 HU), the tumor was separated from the adjacent structure, and the selected ROI was precisely adjusted to the tumor margin. Each ROI was drawn around the gross tumor volume avoiding bone, fat and air. A radiologist with more than 15 years of experience in chest radiology examined all segmented images and selected the best segmentation for data analysis. After drawing the ROIs, AK software (version 3.2.0; GE Healthcare) was used to extract 197 radiomic features (42 histogram, 11 Gy level size-zone matrix (GLSZM), and 144 Gy level cooccurrence matrix (GLCM) features).

### Statistical analysis

#### Important feature selection

A total of 197 texture features were included in this study, among which the measurement data conforming to normal distribution were compared by independent sample t-test; Mann Whitney U tests are used for comparison of measurement data with non-normal distribution. Then, univariate logistic regression was applied to explore whether the features were discriminate between two groups, and minimum redundancy and maximum correlation (mRMR) algorithm was used for feature dimensional reduction to eliminate redundant and irrelevant parameter. Furthermore, multivariable logistic regression analysis is used to select meaningful texture parameters to establish the final radiomics model. Finally, the radiomic score (Rad-score) was constructed using the following formula: Rad-score = intercept + Σβi·Xi.

#### Prognostic model establishment

For categorical variables, the differences in age, sex, tumor diameter (> 5 cm or < 5 cm), T stage, N stage, lobes, burr pleural depression, leaflet, histology, and postoperative adjuvant therapy were analyzed using χ2 test. Univariate and multivariate logistic analyses were used to identify clinical risk features, and by incorporating the radiomics score (Rad-score) and clinical risk features with multivariate Cox regression, a combined model was built. The patients were classified into high-risk and low-risk groups based on the model, with a cut-off value of the median score. The log-rank test was applied to compare the two KM survival curves. The Harrell's concordance index(C-index) was used to evaluate the accuracy of the combination model by evaluating the consistency between the prediction results of the model and the actual observation results.

#### Evaluation and validation of the nomogram

To evaluate the clinical value of the combined model for predicting the 5-year survival rate of patients, decision curve analysis (DCA) was performed to quantify the net benefits across a range of threshold probabilities. In this study, 100-fold leave-group-out cross-validation (LGOCV) was performed to verify the reliability of our results. Then a nomogram for the model was built to provide a more direct way to determine the 5-year OS rates. The prognostic performance of the nomogram was assessed using a calibration plot.

The SPSS software (version 21.0) and R platform (version 3.5.1, www.r-project.org) were used for data analyses. The reported statistical significance levels were all two-sided, and a *P* value of < 0.05 indicated statistical significance.

## Results

### Clinical parameters of the patients

The patient characteristics are summarized in Table 1. Finally, 73 patients were recruited for this study cohort (Fig. 1). There were 28 women (38%) with a mean age of 66 ± 9.0 years (range, 44–85 years) and 45 men (62%) with a mean age of 68 ± 9.3 years (range, 41–85 years). The distribution of TNM staging was as follows: T1-2 was found in 58 patients (79%), T3-4 in 15 patients (21%), N0 in 44 patients (60%), and N1-3 in 29 patients (40%). The tumors were markedly characterized by different sizes, with a mean diameter of 30.4 ± 11.8 mm (range, 10–74 mm). Tumors with burr, leaflet and pleural indentation accounted for 67.12%, 80.82%, and 56.16%, respectively. There are 30 (41%) adenocarcinoma and 43 (59%) squamous cell carcinoma. 15 patients (20.55%) received adjuvant treatment after surgery, and 58 patients (79.45%) did not receive adjuvant treatment after surgery. For OS, the mean survival time was 47.8 months (range, 1.8–60 months).

**Table 1:** Clinicopathological characteristics of patients with non-small cell lung cancer

Characteristics	N	Percentage (%)
Gender		
Male	45	61.64
Female	28	38.36
Age		
> 60	47	64.38
≤ 60	26	35.62
T stage		
T1-2	58	79.45
T3-4	15	20.55
N stage		
N0	44	60.27
N1-3	29	39.73
Tumor size(cm)		
> 5	15	20.55
≤ 5	58	79.45
Burr		
Yes	49	67.12
No	24	32.88
Leaflet		
Yes	59	80.82
No	14	19.18
Pleural indentation		
Yes	41	56.16
No	32	43.84
Histology		
adenocarcinoma	30	41.10
squamous cell carcinoma	43	58.90
Postoperative adjuvant therapy		
Yes	15	20.55
No	58	79.45

### Important radiomic feature selection

**Fig. 2:**
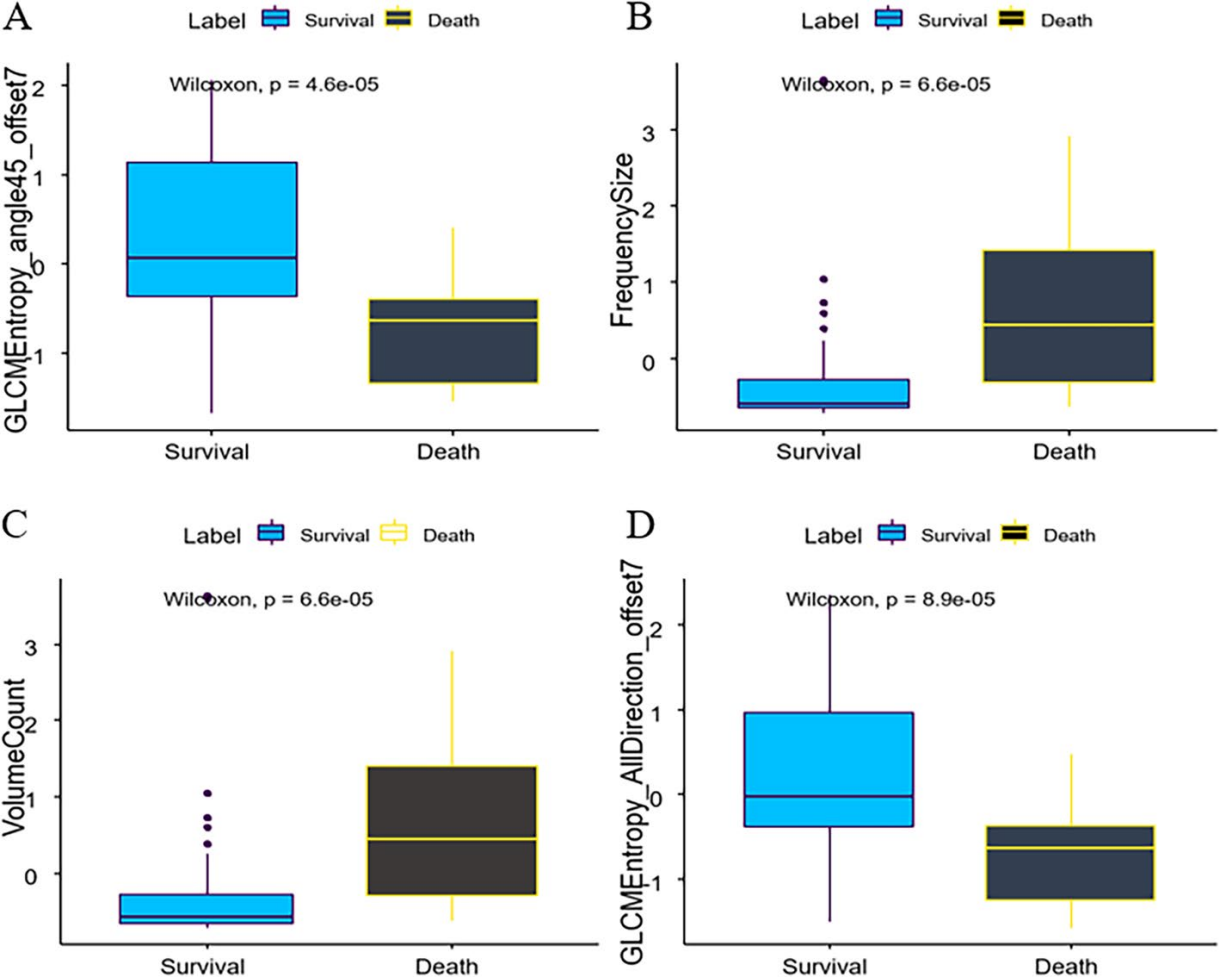
Distribution of significant features associated with survival. The features are listed with their name and statistical significance

**Fig. 3:**
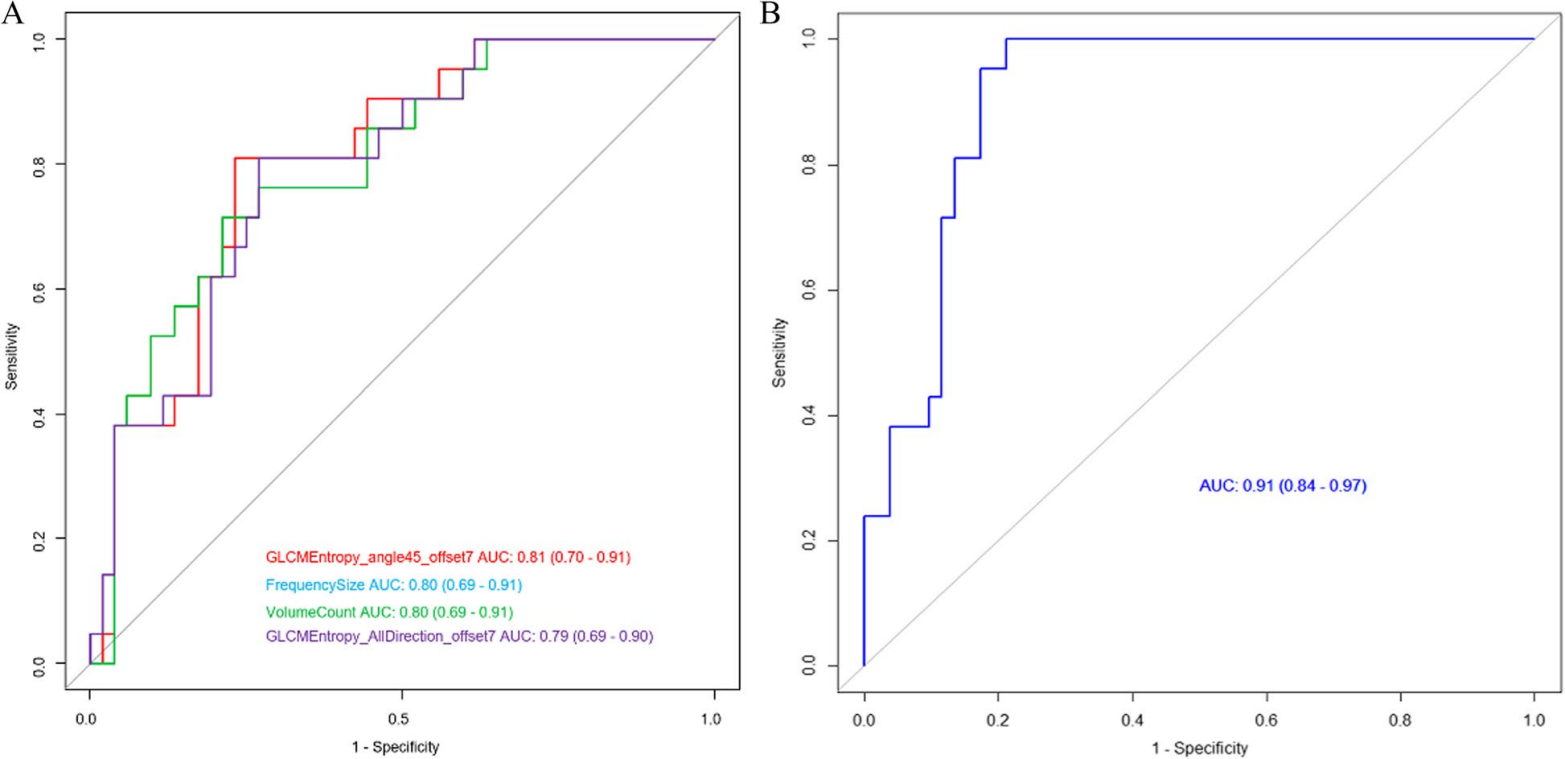
ROC curves of the single texture features (**A**) and integrated model for overall survival prediction

According to independent samples t-test or the Mann–Whitney U test, 95 texture parameters were associated with patient survival (Supplementary file 1). The top 20 features with *P*-values are detailed in Table 2, where the features with the top 4 *P*-values are shown in Fig. 2. Furthermore, 87 features were significant using logistic single factor analysis (Supplementary file 2). The top 20 features with *P*-values are detailed in Table 3. The ROC curves of the top 4 features are shown in Fig. 3A; the AUCs of these parameters were approximately 0.81, 0.80, 0.80 and 0.79. Finally, based on backward stepwise analysis, the remaining features were used for multiple logistic regression analysis to select the optimal parameter for building the integrated radiomic model. We obtained the nine most critical imaging radiomics characteristics, and their OR values are shown in Table 4.

**Table 2:** Comparison of CT texture parameters with independent samples t-test or the Mann–Whitney U test

lower0	median0	upper0	lower1	median1	upper1	*p*_val	VarName
-0.36585	0.07827	1.132055	-1.33341	-0.62833	-0.39176	4.58E-05	GLCMEntropy_angle45_offset7
-0.6556	-0.57668	-0.28017	-0.30021	0.450925	1.411768	6.58E-05	FrequencySize
-0.65559	-0.57668	-0.28017	-0.30021	0.450925	1.411768	6.58E-05	VolumeCount
-0.37648	-0.01503	0.973974	-1.24907	-0.6275	-0.36131	8.94E-05	GLCMEntropy_AllDirection_offset7
-0.37363	-0.03078	1.157748	-1.1766	-0.61167	-0.36641	1.04E-04	GLCMEntropy_angle135_offset7
-0.74581	-0.58818	-0.11325	-0.24689	0.474184	1.259742	1.88E-04	SizeZoneVariability
-0.45714	-0.01546	0.844046	-1.20927	-0.62687	-0.32331	2.17E-04	GLCMEntropy_angle0_offset7
-0.42147	-0.01945	1.101531	-1.18449	-0.61598	-0.32051	2.39E-04	GLCMEntropy_angle90_offset7
-0.29279	0.035278	0.845678	-1.1524	-0.52207	-0.25378	2.63E-04	GLCMEntropy_AllDirection_offset4
-0.28455	0.042619	0.842854	-1.1157	-0.51823	-0.22614	3.17E-04	GLCMEntropy_angle90_offset4
-0.27167	0.04504	0.832255	-1.10302	-0.50097	-0.22386	3.65E-04	GLCMEntropy_angle0_offset4
-0.39446	0.026612	0.838308	-1.13277	-0.50339	-0.26112	5.26E-04	GLCMEntropy_angle135_offset4
-0.76875	-0.61764	-0.01082	-0.225	0.287018	1.153372	5.26E-04	IntensityVariability
-0.32476	0.003628	0.857002	-1.17301	-0.5376	-0.24765	5.51E-04	GLCMEntropy_angle45_offset4
-0.46477	0.137388	0.61404	-1.14682	-0.59047	-0.26883	8.98E-04	GLCMEntropy_angle135_offset1
-0.46887	0.111241	0.608756	-1.02059	-0.5392	-0.36422	9.38E-04	GLCMEntropy_angle90_offset1
-0.46255	0.129024	0.59211	-1.05249	-0.60901	-0.35031	0.001164	GLCMEntropy_AllDirection_offset1
-0.50495	0.110698	0.616434	-1.11619	-0.551	-0.30631	0.001164	GLCMEntropy_angle45_offset1
-0.91848	-0.53911	0.330512	-0.38983	0.810499	1.504898	0.001215	InverseDifferenceMoment_angle0_offset7
-0.86073	-0.5275	0.26296	-0.33111	0.792479	1.395615	0.001323	InverseDifferenceMoment_angle0_offset4

**Table 3:** *P* values of univariate logistic regression analysis of CT texture parameters that were significant in the independent samples t-test or Mann–Whitney U test

2.50%	97.50%	OR	*p*val	VarName
0.07162	0.449704	0.201469	0.000549	GLCMEntropy_angle45_offset7
1.360035	4.466686	2.326262	0.004735	FrequencySize
1.360034	4.466684	2.326261	0.004736	VolumeCount
0.076445	0.463686	0.209915	0.000604	GLCMEntropy_AllDirection_offset7
0.075557	0.478155	0.214445	0.000942	GLCMEntropy_angle135_offset7
1.444737	4.544426	2.451569	0.001838	SizeZoneVariability
0.07752	0.488757	0.218272	0.001078	GLCMEntropy_angle0_offset7
0.084585	0.521672	0.235176	0.001679	GLCMEntropy_angle90_offset7
0.117432	0.581562	0.285983	0.001937	GLCMEntropy_AllDirection_offset4
0.119112	0.593985	0.291138	0.002366	GLCMEntropy_angle90_offset4
0.120316	0.593597	0.291431	0.002249	GLCMEntropy_angle0_offset4
0.127344	0.61219	0.306453	0.002782	GLCMEntropy_angle135_offset4
1.443191	4.546284	2.450443	0.001875	IntensityVariability
0.119564	0.592711	0.292803	0.002325	GLCMEntropy_angle45_offset4
0.117309	0.612743	0.292685	0.003428	GLCMEntropy_angle135_offset1
0.108547	0.596189	0.279507	0.003183	GLCMEntropy_angle90_offset1
0.11348	0.607899	0.287757	0.003461	GLCMEntropy_AllDirection_offset1
0.118819	0.621738	0.297046	0.003881	GLCMEntropy_angle45_offset1
1.432351	4.568538	2.466571	0.002001	InverseDifferenceMoment_angle0_offset7
1.438927	4.602588	2.479454	0.001927	InverseDifferenceMoment_angle0_offset4

**Table 4:** List of odds ratios and *P*-values of significant radiomic features prognostic of overall survival

2.50%	97.50%	OR	*p*val	VarName
0.002838	0.208448	0.035943	0.002269	(Intercept)
1.54E-10	0.593901	3.26E-05	0.062313	GLCMEntropy_angle45_offset7
1.563021	87.05005	9.043365	0.027662	MaxIntensity
2.74E-05	0.296342	0.005247	0.023878	Inertia_AllDirection_offset1_SD
0.830336	15.70424	3.116149	0.122664	HighIntensityLargeAreaEmphasis
1.064645	7.212186	2.533245	0.049333	SizeZoneVariability
0.858355	6.639718	2.266245	0.105909	Correlation_angle0_offset7
1.459625	261.0954	14.85109	0.03751	HaralickCorrelation_angle90_offset1
0.001579	0.563118	0.040797	0.030443	Range
0.144698	55,225,380	1336.04	0.144727	GLCMEntropy_angle135_offset7

### Construction of radiomics signature and prediction model and calculation of efficiency

The Rad-score was constructed according to the following formula: Radscore=-3.32582911330918*(Intercept)+-10.3308190312763*GLCMEntropy_angle45_offset7+2.20203137947982*MaxIntensity+-5.25014679863344*Inertia_AllDirection_offset1_SD+1.13659783895495*HighIntensityLargeAreaEmphasis+0.929501117562691*SizeZoneVariability+0.81812412384377*Correlation_angle0_offset7+2.69807322936027*HaralickCorrelation_angle90_offset1+-3.1991457272293*Range+7.19746493772736*GLCMEntropy_angle135_offset7. The radiomics signature was constructed and showed an AUC of 0.91 (95% CI: 0.84 ~ 0.97, Fig. 3B), which is significantly higher compared to single texture parameters.

Considering that the texture analysis was only based on the training cohort and no verification cohort was used, 100-repeated verification analysis was performed using the boot632 resampling method. The results showed favorable predictive efficacy with a sensitivity of 0.98, and a specificity of 0.99, and an accuracy of 0.85 (Supplementary file 3).

### Development of an individualized nomogram

**Fig. 4:**
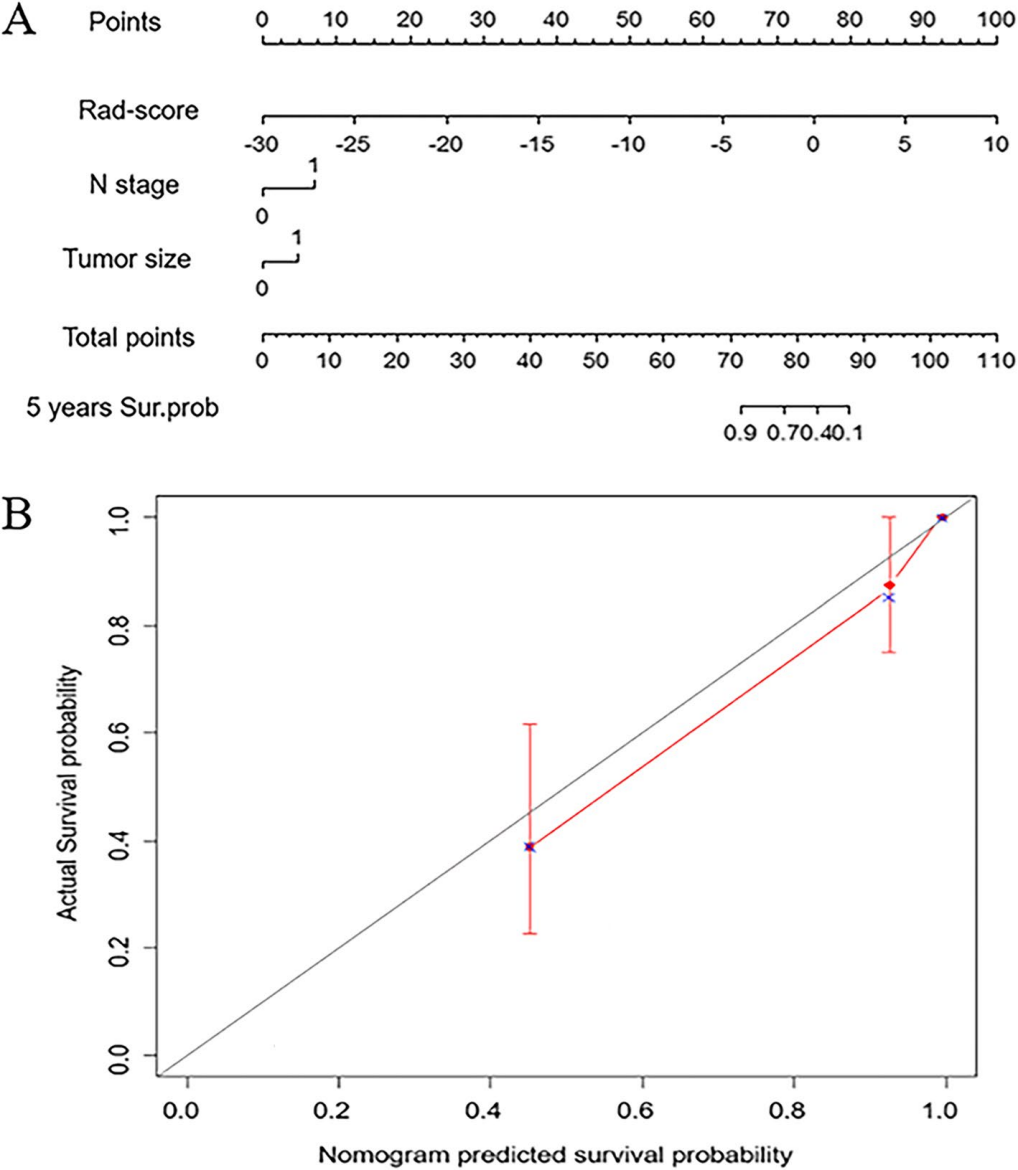
Nomogram for the prediction of overall survival and the calibration evaluation of the model (**A**). The calibration curve of the nomogram predicted the survival probability of OS (**B**)

The clinical risk features were identified by univariate and multivariate logistic analyses (Table 5). Furthermore, multivariate Cox regression analysis revealed that three independent predictors consisting of N stage (HR: 4.54, 95% CI: 1.67–12.38, *P* = 0.003), radiomics model (HR: 1.56, 95% CI: 1.23–1.97, *P* < 0.001), and tumor size (HR: 3.19, 95% CI: 1.29–7.88, *P* = 0.01) were independent predictors of 5-year OS (Table 6). These predictors were used to develop the final model, which was presented as a nomogram (Fig. 4A). The C-index of the model for OS was 0.91 (95% CI: 0.86–0.95, Fig. 4B).

**Table 5:** Univariate logistic and multivariate logistic regression analyses

	Univariate Logistic Regression		Multivariate Logistic Regression	
Variable and Intercept	OR (95% CI)	*P* Value	OR (95% CI)	*P* Value
Age (years)	3.117(0.990–11.992)	0.07	NA	NA
N stage(N0/N1-3)	6.786(2.292–22.423)	< 0.001	647.263(10.385–3,399,898)	0.03*
T stage(T1-2/T3-3)	12.000(3.330–51.500)	< 0.001	NA	NA
Tumor size(cm)	8.545(2.532–32.523)	< 0.001	51.767(2.348–7288.189)	0.04*
Burr	3.750(1.092–17.468)	0.054	301.49(8.46–182,185)	0.02*
Leaflet	7.368(1.329–138.302)	0.062	782.66(4.85–1,434,621)	0.05
Pleural	6.274(1.998–24.231)	0.003*	NA	NA
Histology(adenocarcinoma/squamous cell carcinoma	1.911(0.685–5.328)	0.216	NA	NA
Postoperative adjuvant therapy(Yes/No)	1.493(0.434–5.132)	0.525	NA	NA
Rad-score			10.105(2.249–194.863)	0.03*

^*^P < 0.05

**Table 6:** Multivariate cox proportional hazard regression analysis

Variable	Hazard Ratio	95% Confidence Interval	*P* Value
Rad-score	1.56	[1.23;1.97]	< 0.001*
N stage	4.54	[1.67;12.38]	0.00311*
Tumor size	3.19	[1.29;7.88]	0.01182*
Burr	3.15	[0.83;11.93]	0.09073
Leaflet	3.78	[0.42;33.76]	0.2337

^*^P < 0.05

### Clinical use

**Fig. 5:**
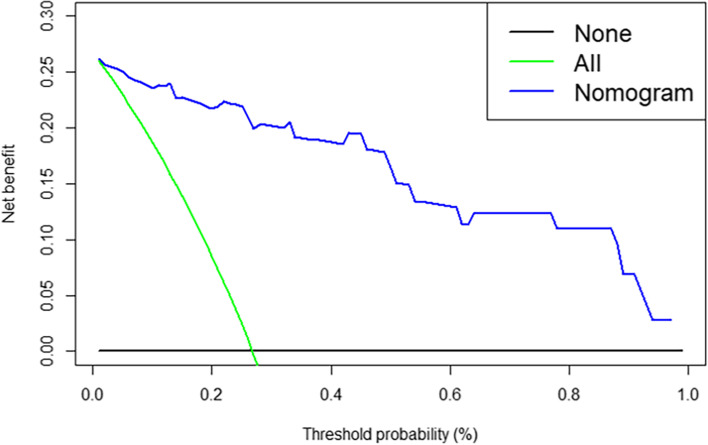
DCA for the radiomics nomogram. The y-axis represents the net benefit, and the x-axis represents the threshold probability. The net benefit of the model is greater than that of the other two cases within the threshold range of 0.1–1, and the prediction using the model is relatively safe

The DCA for the radiomics nomogram demonstrated that the threshold probability of a patient was between 0.01 and 1 (Fig. 5), indicating that the radiomics nomogram might be more beneficial for the prediction of OS.

### Survival analysis

**Fig. 6:**
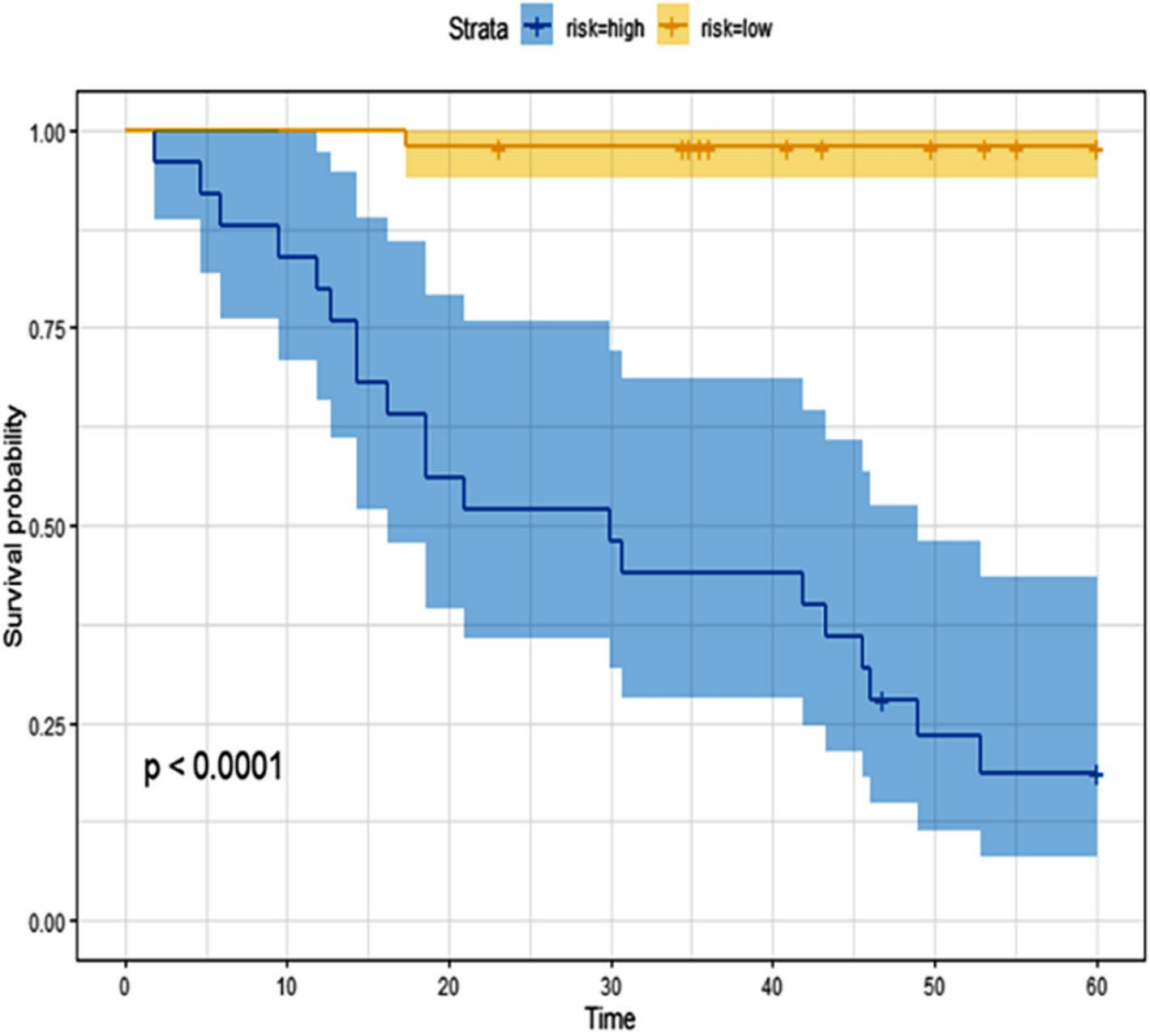
Kaplan–Meier curve showing the overall survival of patients

In addition, to verify the performance of the nomogram, patients were divided into high-risk groups and low-risk groups according to according to the nomogram score. KM analysis showed that higher values of the nomogram were associated with poorer survival (Fig. 6).

## Discussion

The accurate prediction of a patient's prognosis is essential to guide treatment decisions for the patient. In the current study, we used high-throughput data feature extraction algorithms to extract stable and beneficial radiomics features and combined radiomics features and clinicopathological variables to construct a comprehensive predictive model to predict 5-year survival before initial treatment in patients with NSCLC. According to the nomogram score, we further divided all patients into high- and low-risk groups. The KM curves indicated that higher nominal values are associated with poor survival.

At present, the quantitative texture analysis of CT images has attracted attention as a potential method that has the ability to maximize the information obtained from CT images and provide more accurate and comprehensive lesion characterization [15–18]. The heterogeneity of tumors has been found to be closely related to tumor hypoxia and angiogenesis in NSCLC [12, 19, 20]. Furthermore, tumor heterogeneity can be a predictor of patient outcomes [21].

At present, the results showed that CT-based radiomics features have potential predictive value for overall survival in NSCLC patients. GLCM is a correlation function between different gray levels of an image at a specific angle and distance, reflecting the two-dimensional statistical characteristics of the image texture. GLSZM is also a counting matrix, which stores the size and number of connected domains of all gray scales in the image [22, 23]. In our study, among these selected key texture features, GLCM and GLSZM features play an important role in reflecting the heterogeneity of tumor texture. According to the correlation coefficient of histological characteristics, it is found that the entropy and correlation in GLCM based on CT images are significantly more effective in predicting the 5-year survival rate of patients. The entropy mainly reflects the irregularity and complexity of intra-image voxels; the higher the entropy value is, the shorter the survival time. At the same time, correlation is one of the GLCM features that measures the linearity of the images and reflects the similarity GLCMs in the columns or rows. SU et al. [24] reviewed and analyzed the relationship between enhanced CT image texture features and survival in patients with concurrent radiotherapy and chemotherapy for NSCLC and found that higher entropy was an independent prognostic factor for the decline of OS in 3 years (*P* = 0.04). An important study described that GLCM correlation measures the linear dependence of gray levels, which is a key feature for predicting the pathological response in NSCLC [25]. Compared to previous findings, the AUC of our nomogram was higher, which might be explained by the method we used in model building or the clinical features included in our model.

Previous evidence has shown the prognostic value of clinical parameters in NSCLC patients, such as age, maximal tumor diameter, pathological features (T stage, N stage), and morphological features (burr sign, leaflet sign, pleural depression). This study found that N stage is an independent prognostic factor for NSCLC patients, suggesting that the 5-year OS of patients with lymph node metastasis is significantly shorter than that of patients without lymph node metastasis (*P* < 0.05), which is similar to previous studies [26]. The study by Port et al. [27] showed that 244 patients with stage IA lung cancer had a 5-year survival rate of 71.1%, of which 83 patients with a diameter > 2.0 cm ad a 5-year survival rate of 60.3%, which was lower than that of 161 patients with a diameter ≤ 2.0 cm (77.2%) (*P* = 0.03). Christian et al. [28] analyzed the prognostic factors of 548 patients with stage I NSCLC, and found that age was one of the independent factors affecting prognosis and that the prognosis of patients less than 60 years old was significantly better than that of patients older than 60 years old. Nevertheless, in this study, patient age was not a factor influencing NSCLC prognosis by univariate analysis (*P* = 0.099), which may be due to local regional differences. Harpole et al. [29] proposed that visceral pleural involvement is a significant adverse prognostic factor, with a 5- and 10-year survival of 44% and 37%, respectively, compared with the 5- and 10-year survival of patients without pleural involvement (67% and 62%, respectively).

The combined analysis of multiple markers as a signature, rather than individual analyses, is the most promising way to influence clinical management. Thus, in our study, we used high-throughput data feature extraction algorithms to extract specific radiomics features and construct survival prediction models in combination with radiomics features and clinical variables to predict postoperative OS in patients with NSCLC. The data show that the Rad-score based on CT texture features, N stage, and tumor maximum diameter is an independent predictor of OS in NSCLC patients after surgery. Compared with previous studies, the predictive nomogram developed in our study not only is a simple combination of radiomic features but also shows an association between intratumoral heterogeneity and clinical variables with the same function, which is consistent with the current trend of individualized precision treatment. More importantly, we used a variety of techniques to improve the robustness and stability of the texture features and prediction model compared to other models with fewer techniques.

In addition, we further used multifactorial Cox regression to build survival models, and performed KM analysis, and the results showed that the survival of high-risk patients was worse than that of the low-risk group. To verify the clinical application value of the model, we used DCA to show that the threshold value was in a large range of 0.1 ~ 1, and the net benefit of the model was the largest, which indicating that the radiomics nomogram might be more beneficial for individualized treatment and OS prediction of NSLCL patient. For high-risk patients, postoperative adjuvant treatment can be taken to improve the survival prognosis of patients; For low-risk patients, over-treatment can be avoided after surgery, and long-term follow-up can be carried out without adjuvant treatment.

There are some limitations in the present study. First, this study was a single-institutional, retrospective study. Second, as a retrospective study, in some cases, the quality of CT imaging may not be satisfactory. Thus, prospective studies to control confounding factors are expected to be performed and would improve the study design. Third, our study used internal software, so we would like to extend this work to larger cohort and multicenter studies to obtain more clinical results and texture data.

## Conclusion

In conclusion, this study reported that texture features, routine imaging features, and clinical features of patients were strong predictors of survival prognostication for NSCLC patients, and the combination of the three could improve the accuracy of the prediction model more powerfully. This model may help clinicians make better clinical decisions to reduce risk and improve the survival and the quality of life of advanced NSCLC patients.

## Supplementary Information

**Additional file 1.**

**Additional file 2.**

**Additional file 3.**

## Data Availability

The datasets during and/or analyzed during the current study available from the corresponding author on reasonable request.

## Abbreviations

CT: Computed tomography
NSCLC: Non-small cell lung cancer
Rad-score: Radiomics score
C-index: Concordance index
KM: Kaplan–Meier
GLCM: Gray level cooccurrence matrix
OS: Overall survival
ROI: Region of interest
3D: Three-dimensional
GLSZM: Gray level size-zone matrix
DCA: Decision curve analysis
LGOCV: Leave-group-out cross-validation
