# Supplementary material for: Prognostic predictors of radical resection of stage I-IIIB non-small cell lung cancer: the role of preoperative CT texture features, conventional imaging features, and clinical features in a retrospectively analyzed
Source: BMC Pulm Med. 2023 Apr 14;23:122. doi: 10.1186/s12890-023-02422-7 (PMC10105471; doi:10.1186/s12890-023-02422-7)
Supplement: Supplementary file 3 — Additional file 3. [file 12890_2023_2422_MOESM3_ESM.docx]

| Accuracy | Sensitivity | Specificity | Group |
| --- | --- | --- | --- |
| 0.988767 | 0.986379 | 0.994271 | Training |
| 0.858219 | 0.986379 | 0.994271 | Test |

**TableS1:** Sensitivity，specificity, and accuracy of model validation
